# Implementation of a programmatic assessment model in radiation oncology medical physics training

**DOI:** 10.1002/acm2.14354

**Published:** 2024-04-15

**Authors:** Cathy Barbagallo, Kristy Osborne, Claire Dempsey

**Affiliations:** ^1^ Australasian College of Physical Scientists and Engineers in Medicine (ACPSEM) Sydney New South Wales Australia; ^2^ Department of Radiation Oncology Alfred Health Prahran Victoria Australia; ^3^ Australian Council for Educational Research Education Research Policy and Development Division Camberwell Victoria Australia; ^4^ Department of Radiation Oncology Calvary Mater Newcastle Waratah New South Wales Australia; ^5^ Department of Radiation Oncology University of Washington Seattle Washington USA; ^6^ School of Health Sciences University of Newcastle Callaghan New South Wales Australia

**Keywords:** assessment, medical physics education, training

## Abstract

**Purpose:**

In 2019, a formal review and update of the current training program for medical physics residents/registrars in Australasia was conducted. The purpose of this was to ensure the program met current local clinical and technological requirements, to improve standardization of training across Australia and New Zealand and generate a dynamic curriculum and programmatic assessment model.

**Methods:**

A four‐phase project was initiated, including a consultant desktop review of the current program and stakeholder consultation. Overarching program outcomes on which to base the training model were developed, with content experts used to update the scientific content. Finally, assessment specialists reviewed a range of assessment models to determine appropriate assessment methods for each learning outcome, creating a model of programmatic assessment.

**Results:**

The first phase identified a need for increased standardized assessment incorporating programmatic assessment. Seven clear program outcome statements were generated and used to guide and underpin the new curriculum framework. The curriculum was expanded from the previous version to include emerging technologies, while removing previous duplication. Finally, a range of proposed assessments for learning outcomes in the curriculum were generated into the programmatic assessment model. These new assessment methods were structured to incorporate rubric scoring to provide meaningful feedback.

**Conclusions:**

An updated training program for Radiation Oncology Medial Physics registrars/residents was released in Australasia. Scientific content from a previous program was used as a foundation and revised for currency with the ability to accommodate a dynamic curriculum model. A programmatic model of assessment was created after comprehensive review and consultation. This new model of assessment provides more structured, ongoing assessment throughout the training period. It contains allowances for local bespoke assessment, and guidance for supervisors by the provision of marking templates and rubrics.

## 1 INTRODUCTION

The Australasian College of Physical Scientists and Engineers in Medicine (ACPSEM) was formed in 1977 after many years associated with the UK Hospital Physicists Association.^1^ By 1985, the ACPSEM Council determined a need for a formal qualification for practicing medical physicists and introduced an accreditation scheme. The Accreditation in Radiation therapy Equipment Commissioning and Quality Assurance (ARECQA) was established in 1987 and included written, practical, and oral examinations specific to Radiation Oncology Medical Physics (ROMP). Suitably experienced members would undertake this accreditation as a demonstration of their competence in this discipline.

Accreditation did not include a formal syllabus or training program and was not mandated for clinical medical physicists. Candidates were given “on‐the‐job” training with no standardization on the scope or depth of training. It was not until a federal inquiry into Australian Radiation Oncology services in 2002 that a formal national ROMP training scheme was recommended.^2^ This recommendation led to the development of the ACPSEM ROMP “Training, Education and Accreditation Program” (TEAP) for Australia and New Zealand. This acronym has been subsequently modified to “Training, Education and Assessment Program.” With the introduction of TEAP, a certification process was formalized to employ residents (or “registrars”) specifically into training positions. These positions were often funded by state or federal governments and were contractual for the length of the training.

There are numerous publications highlighting the training of medical physicists in different regions.^3^, ^4^, ^5^, ^6^ These publications concentrate on the differences in the intake of candidates into the program, length and structure of the program, and the curriculum covered.

The ACPSEM ROMP TEAP was designed to produce competent, safe‐to‐practice Radiation Oncology Medical Physicists that have the skills and knowledge required to work independently in a radiation oncology department. The clinical training component of ROMP TEAP is 3 years in addition to the time required to complete any required post‐graduate university study (minimum MSc in Medical Physics). Entry into the ACPSEM ROMP TEAP is based on fixed eligibility criteria and selection tools, with clinical training occurring at an ACPSEM accredited clinical training site under the management of an ACPSEM approved supervisor. All registrars across Australasia would be enrolled into the same program and complete the same competencies, regardless of which clinical department they were based at.

The original training content for the ACPSEM ROMP TEAP was developed in 2003 and originally centered around the educational requirements of a medical physicist defined in International Commission on Radiological Protection publication 44^7^ and American Association of Physicists in Medicine (AAPM) Report 36^8^ under the guidance of consultant educational experts. The ACPSEM ROMP TEAP subsequently formed the core of the initial International Atomic Energy Agency (IAEA) ROMP training course.^9^ Since that time, changes in technology and treatment techniques have required updates to some scientific content within the program. Earlier iterations of the ACPSEM TEAP (Versions 1 to 3.6) for ROMPs structured the program around major units of work (modules), and then covered a variety of learning outcomes on topics within each module. Each learning outcome had prescribed assessment criteria, which was assessed by a local clinical supervisor to evaluate candidate competence. The program suggested a range of assessment methods that could be used, but the type of assessment used was at the discretion of the supervisor.

It was noted on the 10th anniversary of the introduction of the ACPSEM TEAP,^10^ that the program was based on workplace competency assessment performed by local clinical supervisors during the training period, with formal external assessment by ACPSEM‐appointed examiners to achieve certification in the final exams. The formal assessment included a written examination after completion of a sub‐set of the curriculum, as well as both practical and oral examinations at the end of the training period. Examinations were conducted by experienced clinical medical physicists. The written examination was independently blind‐marked by two examiners per subject area, and the practical and oral examinations were conducted on‐site in the candidate's department by two independent examiners, requiring a consensus decision.

The biggest hurdle to robust and standardized clinical assessment throughout the training process, was the experience and training of the local clinical supervisors. Some supervisors lacked experience as educators and had not been adequately trained to provide appropriate supervision. Supervisors with limited teaching or training skills would often rely on methods that personally suited them and would use the same methods for all learning outcomes. Because of the wide range of suitable assessment types listed for a particular learning outcome, there was often a large disparity in the level of assessment used between registrars from different clinical departments. The range in competency evidence submissions from the cohort of registrars across Australia and New Zealand indicated a lack of a cohesive assessment standard being applied.

Programmatic assessment spreads measurement of performance across a range and variety of assessment methods during the training process.^11^ The design and effectiveness of the assessment program as a whole is emphasized, rather than focusing on the adequacy of individual assessments of performance. This is because a program of assessment recognizes that assessing complex competencies requires a range of measures over time and cannot be adequately learned and assessed through a single, point in time assessment.^12^

Although programmatic assessment approaches have become highly regarded in health profession education, this approach contrasts significantly with traditional summative, mastery‐based approaches to assessment and learning. The major shift required to embed a programmatic assessment approach in a training program means that implementation is often challenging.^13^, ^14^ For example, the traditional formative/summative dichotomy is replaced with a continuum of stakes, from low‐ to high‐stakes with a wide variety of assessment tools. This allows the learner to demonstrate growing depth and breadth of knowledge in a discipline.^15^ Each individual assessment datapoint then contributes to the evidence base for determining clinical competence.

In 2019, the ACPSEM initiated a project to update the existing training program^16^ with the requirements that it would reflect the current needs in training for radiation oncology medical physics. This included a dynamic curriculum that was able to be adaptive to changing technologies.

In addition, the curriculum needed to be able to be delivered anywhere within Australasia, allowing for limited equipment access in smaller remote and regional departments. Finally, the clinical training component needed to be completed within a 3‐year time frame, as mandated by the federal funding bodies providing financial support for the program. Over‐arching this was a requirement that it also comply with the Australian Medical Council (AMC)^17^ and Australian Health Practitioner Regulation Agency (AHPRA) standards.

## 2 METHOD

The renewal of the ACPSEM ROMP TEAP officially commenced in early 2020 and proceeded through several key phases to reach implementation. Phase 1 included an expert medical training consultant desktop review of the program, incorporating an analysis of trends and identifying gaps in terms of AMC standards. It also included key stakeholder consultation with targeted online and phone‐based questionnaires. In phase 2, the primary project committee consisting of clinically certified and highly experienced medical physicists, medical physics training specialists, and medical education consultants, developed a series of program outcomes statements that would be used to ensure alignment of all content to graduate attributes. In phase 3, working groups were formed by clinically certified ROMP experts with experience in past training programs who reviewed and updated the scientific content of the training program.

Finally in phase 4, an assessment committee of clinically certified and highly experienced medical physicists, academic medical physicists, and education specialists was formed to create a model of programmatic assessment for the ACPSEM ROMP TEAP. The committee in this phase was led by assessment specialists from the Australian Council for Educational Research (ACER). After determining scope and layout of the new curriculum in phases 2 and 3, a series of suitable assessment methods were discussed, that aligned with the program outcomes, curriculum content, and teaching and learning strategies of the program.^18^

The educational concepts used to underpin the curriculum development, and to inform the assessment methods, were based on the revised Bloom's Taxonomy.^19^ Under this educational model, domains of learning are defined and used as methods of determining breadth and depth of knowledge and skill in content areas. The application used here was divided into cognitive and behavioral domains to both describe the understanding and recall of knowledge, as well as the application of that knowledge clinically.

An assessment method was considered potentially suitable if it were fit‐for‐purpose for the particular learning outcome.^20^ Assessment committee members reviewed all assessment methods and voted on their preferred assessment method for each learning outcome in the new training curriculum framework.

## 3 RESULTS

The key themes to emerge from the consultation in phase 1 included requirements for more standardized methods of assessment and a reduction in the duplication of learning outcomes. From these themes, there were several key recommendations generated:

‐ Clearly identified program outcomes
‐ A review and update of program content (curriculum)
‐ Development of a model of programmatic assessment

In phase 2, a series of program outcome statements were created. These statements were based on the Canadian Medical Education Directives for Specialists (CanMEDS) framework for medical education and practice^21^ and reflected the attributes of graduates of the ROMP TEAP, and their development throughout their professional careers. These traits have been defined under the following categories:

- **Safety**: Works safely within the clinical environment of radiation oncology through the application of evidence‐based practice and risk management in compliance with regulations.
- **Knowledge**: Communicates scientific knowledge effectively and demonstrates skills for the core areas of radiation oncology.
- **Critical thinking/problem solving**: Provides sound radiation oncology medical physics guidance while exercising critical and innovative thinking, problem solving and judgment in a clinical or academic setting.
- **Communication and teamwork**: Communicates and collaborates effectively within a multidisciplinary team ensuring the patient and quality of care is of primary focus.
- **Patient focused**: Practices patient centered radiation oncology medical physics with compassion and respect, using ethical and professional values.
- **Educator**: Provides education, training, and supervision to facilitate the functions of the profession.
- **Continuous Professional Development (CPD)**: Demonstrates commitment to ongoing life‐long professional development and learning.

In phase 3, scientific content in the ACPSEM clinical training guide (v3.6) was assessed by groups of craft ROMP experts for currency. New, emerging techniques and technology were incorporated, and areas of duplication were removed from the curriculum framework. Each learning outcome in the new curriculum was linked to at least one of the program outcome statements, to ensure they were fit for purpose. In the new curriculum, 10 key areas were identified for ROMP clinical training (see Table 1) including one key area assigned for new and emerging technologies. Note that at the time of writing, there are no proton therapy centers in Australia, and MRI Linacs are extremely limited. As such, they are not considered core curriculum content, but have been included as part of the emerging technologies in the Australasian context.

**TABLE 1:** The 10 key areas for radiation oncology medical physics training.

No.	Key area	Content description
1	*Clinical Introduction to Radiation Oncology Medical Physics*	Foundations of radiation oncology and the patient experience, as well as the medical physics community within Australasia. Mandatory introductory topic to be completed within first 6 months of training via reflection submissions. Not examinable content.
2	*Radiation Safety and Protection*	Radiation protection legislation, radiation safety and shielding design and survey techniques.
3	*Dosimetry*	Radiation detection systems and their use in absolute and relative dosimetry including conditions of disequilibrium.
4	*Linear Accelerator‐Based Treatment*	Clinical beam production principles, as well as the commissioning and quality assurance required for all linac applications.
5	*MV External Beam Treatment Planning*	Radiobiology, treatment planning systems and the design and evaluation of quality treatment plans.
6	*Superficial and Orthovoltage Therapy*	All aspects of dosimetry, shielding and treatment planning for superficial and orthovoltage treatment energies.
7	*Imaging for Radiation Oncology*	Imaging system principles and operation used for cancer imaging and treatment planning in radiation oncology.
8	*Information and Communication Technology*	Includes Oncology Information Systems, database management, software automation and the use of AI applications in clinical practice. Not examinable content.
9	*Brachytherapy*	All aspects of dosimetry, radiation safety, QA and treatment planning for low‐ and high‐dose rate brachytherapy.
10	*Advanced Technologies*	Includes particle therapy and MRI linacs. This is also a placeholder for technologies as they are introduced into the market. As they become common practice they can be integrated into the curriculum in their own key area. Not examinable content.

*Note*: Unless noted otherwise, the content is formally examinable.

In addition to the curriculum to be studied, registrars are required to complete the following:

- Three clinical and scientific reports (CaSRs) which are designed to provide evidence of increasing depth of understanding and clinical involvement. Each report is assessed by an external, independent expert reviewer, and the final report is also assessed via an oral examination.
- Online written examinations covering core medical physics content.
- Presentation at a recognized national or international conference on clinical or research work conducted during the period of clinical training.
- Regular performance reviews throughout the program on training and milestone progress conducted by an external, independent expert assessor.
- Oral and practical examinations for final certification.

All (i) to (v) assessment methods were part of the previous curriculum design with slight modifications to timing or delivery methods.

In designing the ACPSEM ROMP TEAP programmatic assessment model in phase 4, many assessment methods were considered. Some of the resulting core assessment methods can also be considered as Structured Learning Activities (SLAs) due to feedback from the assessment providing a learning opportunity for the registrar. The SLAs considered, and their definitions, were as follows:

- Entrustment Activity – Registrars are given increasing levels of responsibility/trust in an ongoing routine task, coupled with decreasing levels of supervision.
- Written Task/Report – A short report outlining the work conducted on a task or understanding of a specific topic.
- Oral Assessment – A structured oral assessment interview on a topic.
- Multiple Choice Question (MCQ) Activity – An app‐based series of questions (from a question bank) that cover the required fundamental content of a topic.
- Practical Assessment – Observation of a practical skill that is not part of routine quality assurance, but forms part of the normal skill set for a clinical physicist.

In deciding the SLA that would provide final evidence for assessment, the assessment committee was asked to vote for which SLA that they each felt would be most appropriate for each specific learning outcome. For some learning outcomes the group were unanimous in their recommendation of the assessment type. For other learning outcomes there were a spread of responses indicating different assessment preferences for each committee member. This often resulted because some learning outcomes lend themselves to multiple ways of assessment due to the nature of the content within them.

Final decisions on individual learning outcome assessment methods were discussed with expert educational consultants to determine the fitness‐for‐purpose and alignment of the assessment method to learning outcome, training opportunities, curriculum, and graduate outcomes.^17^, ^18^ Through these sessions, in cases where there was originally no clear consensus, a majority decision could be found that was satisfactory to all committee members.

On completion of voting and discussion, the final list of SLAs was now mandated as the learning activity (with associated assessment type) to be used for each learning outcome. The final ACPSEM curriculum framework contained 79 different learning outcomes, which were further broken into clarifying elements where required. For 73 learning outcomes, the breakdown of SLAs agreed by the committee was 23 written task/reports, 21 oral assessment, 12 MCQ activities, 12 entrustment activities and five practical assessments. There were also six additional learning outcomes that were tied to online learning modules with automated online assessment. Supplement 1 provides the full list of learning outcomes, elements, and their associated SLAs in the final curriculum.

The aim of the SLAs was to ensure that all registrars are being assessed in the same way and receiving the same learning opportunities. To aid in successful implementation of the progressive assessment model and standardization of assessment, assessment rubrics were created to be used as a tool for assessors for each assessment type in the programmatic assessment model.

Using rubrics as a marking aid assists in providing feedback to registrars on their learning across all domains, as well as providing registrars with clear descriptions of the expected standard.^22^ All rubrics consisted of a three‐point scale (Falls Short / Meets / Exceeds), describing what a learner needs to demonstrate in order to meet the required minimum expectations for the learning outcome. The rubrics have been designed to be useful for a range of assessment types, to unify the language used in assessment, and to assess a range of skill sets so that they are useable throughout the whole program.

All work should be marked against a set of criteria which covers both cognitive and behavioral domains of learning, selected from the following categories:

- Ability to perform practical tasks,
- Clinical medical physics judgment and responsibility,
- Demonstrates critical and thorough scientific thinking,
- Application of relevant theory to clinical situations, and
- Communication.

Table 2 shows an example assessment rubric for a practical activity. In this instance, communication is not used as part of the grading scheme. Registrars are required to achieve the standard, Meets Expectations, in each category to be considered competent. For entrustment‐based activities, which are graded over several levels of trust/responsibility, a registrar falling in the Exceeds Expectations may be considered ready to move to a higher level of entrustment.

**TABLE 2:** Assessment rubric for practical activities.^23^

Criterion	Falls short of expectations	Meets expectations	Exceeds expectations
*Ability to perform practical tasks*	• Demonstrates proficiency in practical tasks but still requires some indirect supervision • Demonstrates ability to independently perform practical tasks, however with some deficiencies such as taking excessive time, asking the supervisor about some routine tasks	• Demonstrates ability to independently perform practical tasks concisely and to a high standard • Demonstrates independent proficiency in practical tasks	• Demonstrates independent proficiency in practical tasks and may be trusted to supervise new Registrars
*Clinical medical physics judgment and responsibility*	• Cannot demonstrate the ability to discuss the clinical importance of results without heavy guidance from the supervisor • Demonstrates the ability to flag unusual results with supervisor, but discussions about these are supervisor‐led • Demonstrates poor clinical judgment in routine work	• Demonstrates the ability to independently discuss the clinical importance of results • Demonstrates the ability to flag and hold a Registrar‐led discussion about unusual results with supervisor	• Demonstrates thorough and independent clinical judgment in both routine work and non‐routine results
*Demonstrates critical and thorough scientific thinking*	• Demonstrates an understanding of most theory, but some weaknesses still present • Demonstrates the ability to use theory to guide clinical practice, but needs guidance from supervisor • Unable to demonstrate justification of procedures and/or equipment	• Demonstrates a strong understanding of all relevant theory • Demonstrates the ability to use theory to guide clinical practice independently • Demonstrates sound justification of all procedures and equipment	• Demonstrates an extensive knowledge of theoretical concepts • Uses innovative thinking to suggest improvements to clinical practice • Demonstrates sound justification of all procedures and equipment and demonstrates justification of some innovative and/or novel equipment
*Application of relevant theory to clinical situations*	• Demonstrates only a basic understanding of why tasks are performed	• Demonstrates an understanding of the rationale and purpose behind all work performed	• Demonstrates the ability to critique routine procedures

Templates for assessment incorporating the assessment rubrics were created and supervisors were encouraged to use these templates to ensure that they are assessing the methods prescribed in the programmatic assessment model.

## 4 DISCUSSION

The project to update the training program for Australasian radiation oncology medical physicists was planned in four phases. These phases covered addressing the overall intended outcome of the program, content of the curriculum, meaningful assessment, and implementation. The key aim of the project was the inclusion of programmatic assessment, with structured learning to address evidence of assessment. In addition, the training was to be broken into three clear stages including Stage A (Foundation), Stage B (Core) and Stage C (Consolidation) with each stage anticipated to take 12 months. Progression between stages can occur at other times, depending on different factors.

Within each stage, there are:

- Hurdle requirements, which must be completed before the registrar is eligible for progression.
- Training and assessment evidence requirements, which must be collated in each stage.
- Ad hoc learning opportunities, which are not mandatory. Examples of ad hoc learning opportunities include (but are not limited to): Tutorials (both in‐house, online and via workshops), patient case studies, departmental projects (e.g., commissioning) and non‐routine quality assurance, informal discussions (with supervisors, trainers, registrars, other multidisciplinary staff, or patients), presentations to physicists, registrars, other multidisciplinary staff or patients.
- Structured Learning Activities (SLAs), which are mandatory. These are specifically mapped to learning outcomes, and satisfactory completion of SLAs (along with any ad hoc learning opportunities) allows the registrar to attain the skills stated in a learning outcome.

**FIGURE 1:**
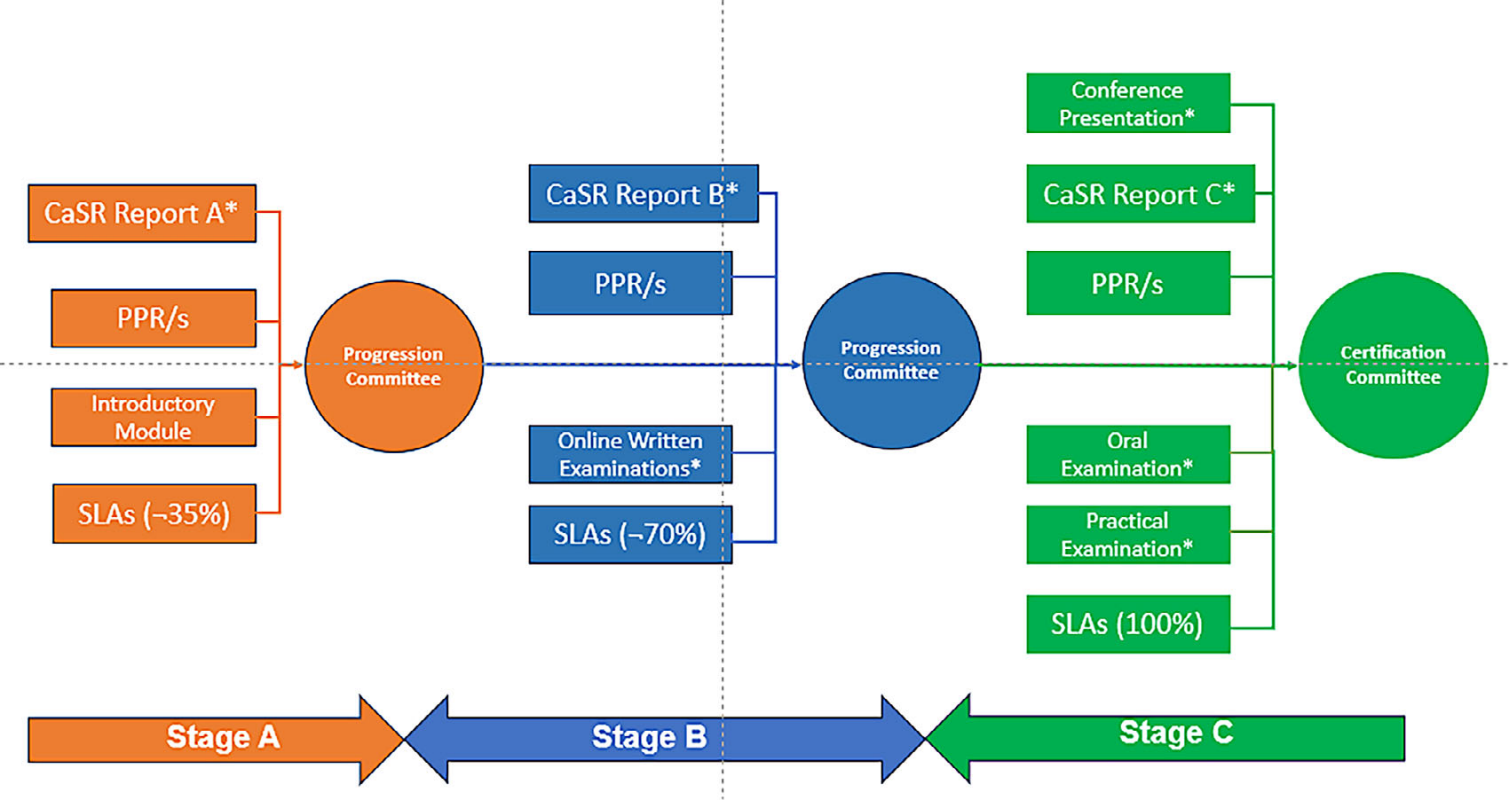
Diagram of progress through the ACPSEM TEAP. Registrars are enrolled into training positions (after satisfying intake requirements) and progress through three stages. After the final examinations and all other hurdle requirements are met, registrars are certified as qualified medical physics specialists. ACPM, Australasian College of Physical Scientists and Engineers in Medicine; TEAP, Training, Education and Accreditation Program.

Figure 1 illustrates the diagrammatic summary of the process from enrollment (prior to Stage A) through to final certification (at Stage C completion). Hurdle requirements are denoted with an asterisk. Note that the proportion of SLAs to be completed in each stage is spread across the key areas, ensuring that the registrars gain knowledge in all disciplines.

Progression from Stage A to B, Stage B to C, and Stage C to completion (Certification) is a high‐stakes decision made by a progression committee. The committee comprises an ACPSEM training coordinator and representatives from the Radiation Oncology Certification Panel who are experienced in training and have extensive knowledge of the ACPSEM TEAP requirements. They review all submitted evidence of training and assessment to make an informed decision of registrar progress in the program. Registrars have flexibility in the attainment of learning outcomes, especially in the order in which they are undertaken. This recognizes the variation in training center programs and contexts.

Management of the curriculum and registrar learning is via an online Learning Management System (LMS). The system allows registrars to upload any training evidence, find resources and keep records on their progress. In addition, supervisors and other medical physicists who perform assessments can provide records of assessment (including assessment templates) and feedback given to the registrar.

An important element of education is the key requirement of providing meaningful feedback and encouraging communication between supervisors and registrars.^15^, ^24^ This allows registrars to enhance their learning and show growth from that feedback.^25^ In each stage of the ACPSEM TEAP, registrars must complete a range of activities and milestones including independent external periodic progress reviews (PPRs).

These reviews are conducted by trained assessors who monitor progress via an interview with the registrar and their supervisor. The registrar will be asked a range of questions on the learning outcomes that they have completed to assess their understanding and competence. Supervisors are also given opportunity to discuss their work with the registrar and any difficulty or success they are having. The outcome of the PPR is graded against the same behavioral and cognitive assessment rubrics, and the interval to the next review is based on their overall performance. Registrars who perform below expectations for their time in the program will be reviewed at either 3‐ or 6‐month intervals. Similarly, registrars who are performing at or above expectations, according to their time in the program, are reviewed at 9‐to‐12‐month intervals.

The new program was released for enrolment in July 2022. At that time, registrars who had been in the previous program (v3.6) for less than 12 months were given the option to transfer to the new program (ROMP2022). Their progress against the new curriculum was determined and they were assigned recognition of prior learning (RPL) in completed learning outcomes. Across Australia and New Zealand, as at July 2022, there were approximately 80 registrars in the ROMP TEAP, of which 65% were enrolled in ROMP2022. While in the current transition phase, all registrars under the V3.6 program will be supported to their completion. All registrars, regardless of program, will use the same rubric marking templates for the external assessments and examinations.

Currently, the program has been in place for 18 months, and initial feedback indicates the registrars are responding well to the change in assessment methods and increased feedback and are now progressing in a timely manner through the program. The main source of outreach and training on programmatic assessment has been centered on the clinical medical physicists who are supervising registrars in their department. The ACPSEM has provided significant training in the form of webinars, presentations at conferences, and feedback through PPRs to assist supervisors with the transition. The largest cause for concern has been noted as an increase in workload for supervisors who are now required to provide greater feedback to registrars than previously. Similarly, the use of rubrics is still a challenging skill that many are adapting to.

The ACPSEM is committed in continuing to provide ongoing training to its members in how to provide high quality feedback to registrars, skills required for conducting assessment (e.g., oral assessment) and ensuring equitable training opportunities and standards are maintained. Future work will assess any further training gaps that need to be addressed, as well as the success of the project overall.

The new curriculum appears to be meeting the current needs of the profession and the introduction of key area 10 (Advanced Technologies) has been a useful tool in allowing the curriculum some dynamic flexibility. There are plans to recognize the increasing use of artificial intelligence in radiation oncology by providing a dedicated space for this in key area 10 in the near future.

## 5 CONCLUSION

Previous assessment for TEAP learning outcomes, and final assessment, left the way in which the work was assessed, and marked, at the discretion of the supervisor or examiner. Now, templates and clear rubrics provide a well‐defined indication of the level expected, while also providing feedback to the learner, on where they may have strengths or weaknesses in the work. Registrars had previously borne the burden of proof of their competency in topic areas, by having to assemble portfolios of work that may or may not display competence. Now the proof of competence is shared with the supervisors in providing rich feedback on the work, areas of improvement, notes taken during observations or practical skills, as well as records of oral assessments and other work assessed collaboratively.

## AUTHOR CONTRIBUTIONS

All listed authors have contributed to the intellectual content, design of the work undertaken and interpretation of data, as well as approval of the published version.

## CONFLICT OF INTEREST STATEMENT

The authors have no conflicts of interest to declare.

## Supporting information

Supporting Information
