# Supplementary material for: Implementation of a programmatic assessment model in radiation oncology medical physics training
Source: J Appl Clin Med Phys. 2024 Apr 15;25(5):e14354. doi: 10.1002/acm2.14354 (PMC11087179; doi:10.1002/acm2.14354)
Supplement: Supplementary file 1 — Supporting Information [file ACM2-25-e14354-s001.docx]

| **Key Area 1 – Clinical Introduction to Radiation Oncology Medical Physics** | | | | | |
| --- | --- | --- | --- | --- | --- |
|  | *Topic 1.1 - Radiation Oncology Medical Physics* | | | | |
|  | | LO 1.1.1 Explain the foundations of Radiation Oncology, including; | | | |
|  | | | E 1.1.1a - The basics of cancer, its diagnosis and treatment  E 1.1.1b - The decision process for cancer management that leads to patients receiving radiation therapy  E 1.1.1c - The aims and effects of radiation therapy in the management of cancer  E 1.1.1d - The patient journey in radiation oncology | Online Assessment |  |
|  | | LO 1.1.2 - Connect the foundations of Medical Physics to a Radiation Oncology setting, including: | | | |
|  | | | E 1.1.2a - Radiation oncology medical physics as defined by the ACPSEM  E 1.1.2b - Medical physicist’s ethical and legal responsibilities  E 1.1.2c - The responsibility associated with working in a clinical setting  E 1.1.2d - The importance of advocating for patient and staff safety and in a wider capacity educate, teach, and communicate radiation safety  E 1.1.2e - The importance of applying professionalism in the workplace  E 1.1.2f - Lines of communication in a department  E 1.1.2g - The organizational structure within the department with respect to other professionals | Online Assessment |  |

| **Key Area 2 – Radiation Safety and Protection** | | | | |
| --- | --- | --- | --- | --- |
|  | *Topic 2.1 - The principles, application and risks of radiation protection* | | | |
|  | | LO 2.1.1 Identify and discuss local radiation protection legislation, including: | | |
|  | | | E 2.1.1a - How local legislation is implemented practically in the department and how it relates to national and international recommendations  E 2.1.1b - Where and how radiation risk is communicated with relation to radiation oncology  E 2.1.1c - The key concepts of radiation protection and role of the Radiation Safety Committee/Officer regarding protection management for an organization | Oral Assessment |
|  | | LO 2.1.2 Understand and practice radiation protection methods, including: | | |
|  | | | E 2.1.2a - Evaluating the risks of radiation-induced damage during radiation therapy treatment  E 2.1.2b - Radiation protection for medical, occupational, public and environmental exposures  E 2.1.2c - Personal monitoring, evaluating results and taking appropriate actions  E 2.1.2d - The importance of advocating for patient and staff safety and in a wider capacity educate, teach and communicate radiation safety | Oral Assessment |
|  | | LO 2.1.3 Explain radiation protection legal compliance, including: | | |
|  | | | E 2.1.3a - The various licenses / registrations / approvals in the department for staff and equipment (as applicable), and the importance of auditing department compliance with regulatory requirements  E 2.1.3b – The legal requirements for personal monitoring | Oral Assessment |
|  | | LO 2.1.4 - Practice and advise on radiation protection, including: | | |
|  | | | E 2.1.4a - The principal requirements of radiation protection management  E 2.1.4b – Evaluating compliance processes in radiation protection  E 2.1.4c - Assessing radiation protection risks in relation to medical, occupational and public exposure to ionizing radiation  E 2.1.4d – Comparing risk information from ethics committees, clinical trial dose and risk assessments for patients undergoing radiation therapy in radiation oncology vs nuclear medicine therapy patients | Written Task or Report |
|  | *Topic 2.2 - Radiation shielding and surveys for linear accelerators* | | | |
|  | | LO 2.2.1 – Understand shielding techniques for linear accelerators, including: | | |
|  | | | E 2.2.1a - The requirements and principles of shielding construction and protection for linear accelerators  E 2.2.1b - The key concepts for shielding construction calculations for linear accelerators  E 2.2.1c - Determining barrier thicknesses and expected exposure levels for linear accelerators | Written Task or Report |
|  | | LO 2.2.2 - Perform radiation surveys and compare to design calculations, including: | | |
|  | | | E 2.2.2a – Selection of appropriate radiation protection instrumentation (e.g. survey meter and dosimeters)  E 2.2.2b - Evaluating survey results and providing recommendations | Practical Activity |
|  | | LO 2.2.3 - Practice and advise on shielding design for linear accelerators, including: | | |
|  | | | E 2.3.1a - Public signage, leaflets, posters, visual and audible alarms, staff training and inductions | Written Task or Report |
|  | *Topic 2.3 - Managing radiation incidents and accidents* | | | |
|  | | LO 2.3.1 - Describe common types of incidents and accidents and recognize prevention methods, including: | | |
|  | | | E 2.3.1a - Public signage, leaflets, posters, visual and audible alarms, staff training and inductions | MCQ Activity |
|  | | LO 2.3.2 - Describe and practice key actions and considerations for radiation incidents and accidents, including: | | |
|  | | | E 2.3.2a - Identifying unsafe situations  E 2.3.2b - The required communication with those involved incidents, including relevant authorities  E 2.3.2c - Determining any dose estimations  E 2.3.2d - Long-term action requirements | Practical Activity |
|  | | LO 2.3.3 - Manage safety and protection in relation to radiation incidents and accidents, including: | | |
|  | | | E 2.3.3a - Judgements and actions to prevent incident recurrence | Practical Activity |

| **Key Area 3 – Dosimetry** | | | | |
| --- | --- | --- | --- | --- |
|  | *Topic 3.1 - Foundation Dosimetry* | | | |
|  | | LO 3.1.1 - Explain the theory of radiation detection and the operation of key detectors, including: | | |
|  | | | E 3.1.1a - The physical principles and operation of ion chambers for MV and kV dosimetry  E 3.1.1b - The physical principles and operation of film for MV and kV dosimetry  E 3.1.1c - The physical principles and operation of diodes for MV and kV dosimetry  E 3.1.1d – The physical principles and operation of EPIDs for MV dosimetry | MCQ Activity |
|  | | LO 3.1.2 - Describe and practice commissioning or QA for detectors, including: | | |
|  | | | E 3.1.2a - Commissioning or QA for an ion chamber  E 3.1.2b - Commissioning or QA for a dosimeter other than an ion chamber | Practical Activity |
|  | | LO 3.1.3 - Explain the theory of dosimetry phantoms and their use, including: | | |
|  | | | E 3.1.3a - The physical principles, operation and use of phantoms | MCQ Activity |
|  | | LO 3.1.4 - Describe and practice commissioning or QA for dosimetry systems, including: | | |
|  | | | E 3.1.4a - Commissioning or QA for water tank dosimetry systems  E 3.1.4b - Commissioning or QA for other phantoms or ancillary components | Practical Activity |
|  | | LO 3.1.5 - Explain the purpose and theory of reference dosimetry, including: | | |
|  | | | E 3.1.5a - How calibration factors are transferred from the PSDL to the department  E 3.1.5c - The theory of key dosimetry protocols  E 3.1.5b - The key principles of the protocol used for absorbed dose determination in the department | Oral Assessment |
|  | | LO 3.1.6 - Describe and practice absorbed dose measurement under reference conditions, including: | | |
|  | | | E 3.1.6a - The radiation quality for MV photons and electrons  E 3.1.6b - The cross calibration of ion chambers  E 3.1.6c - Reference dosimetry under reference conditions | Entrustment Activity |
|  | | LO 3.1.7 - Explain the purpose and theory of non-reference (relative) dosimetry, including: | | |
|  | | | E 3.1.7a - Relative dosimetry theory  E 3.1.7b - Key relative dosimetry terms | MCQ Activity |
|  | | LO 3.1.8 - Explain the theory and measurement techniques of disequilibrium dosimetry, including: | | |
|  | | | E 3.1.8a - Theory of disequilibrium conditions  E 3.1.8b - How dosimetry is performed in disequilibrium conditions  E 3.1.8c - The physical characteristics of the detectors used for small field measurements in the department | Oral Assessment |
|  | | LO 3.1.9 - Clinically apply measurements in conditions of disequilibrium, including: | | |
|  | | | E 3.1.9a - Perform measurements in conditions of disequilibrium | Entrustment Activity |
|  | *Topic 3.2 - In-Vivo Dosimetry* | | | |
|  | | LO 3.2.1 - Explain the purpose and theory of in vivo dosimetry, including: | | |
|  | | | E 3.2.1a - The purpose of an in-vivo dosimetry program  E 3.2.1b - The physical principles of the in-vivo dosimeter used in the department  E 3.2.1c - The uncertainty of in-vivo dose measurements | Oral Assessment |
|  | | LO 3.2.2 - Describe and practice in-vivo dosimetry for the department, including: | | |
|  | | | E 3.2.2a - Performing in-vivo dosimetry measurements for the department  E 3.2.2a – Interpreting and making clinical recommendations based on in-vivo dosimetry measurements in the department | Entrustment Activity |
|  | | LO 3.3.1 - Manage a dosimetry project for your department, including: | | |
|  | | | E 3.3.1a – Completing a department level project from a prescribed list:  Pick one topic suggested below:     1. Manage the commissioning of a new dosimeter 2. Analyze an existing program and make recommendations 3. Manage commissioning of a phantom 4. Develop a new absorbed dose protocol for department 5. Respond to faults and perform tests to return a treatment unit to clinical service after repairs in real or mock scenarios 6. Audit dose for a treatment unit at another department 7. Lead department’s participation in multi-center dosimetry inter-comparisons 8. Work with external regulators during dosimetry audits 9. Commission an in-vivo dosimetry system 10. Manage an in-vivo dosimetry system 11. Provide advice to other professional groups on the suitability of in-vivo dosimetry requests     Review existing in-vivo dosimetry practice with aim of determining its accuracy, precision and clinical utility | Written Task or Report |

| **Key Area 4 – Linear Accelerator-Based Treatment** | | | | |
| --- | --- | --- | --- | --- |
|  | *Topic 4.1 - Linac operation, commissioning and QA* | | | |
|  | | LO 4.1.1 - Explain the operational principles of a linac and the physical principles of clinical beam production, including: | | |
|  | | | E 4.1.1a - The function and operation of the beam forming components  E 4.1.1b - The function and operation of mechanical and optical systems  E 4.1.1c - Linac safety features and regulatory requirements  E 4.1.1d - The physical principles of electron acceleration and transport  E 4.1.1e - The physical principles of X-ray and electron beam formation | MCQ Activity |
|  | | LO 4.1.2 - Explain how beam shaping works for clinical treatment, including: | | |
|  | | | E 4.1.2a - The function and operation of photon beam shaping components  E 4.1.2b - The design and use of Multi-Leaf Collimator (MLC) systems  E 4.1.2c - The function and operation of electron beam shaping components  E 4.1.2d- Other methods of shaping clinical beams and their limitations | MCQ Activity |
|  | | LO 4.1.3 - Explain the attributes and control of clinical beams, including: | | |
|  | | | E 4.1.3a - The features and physical principles of photon and electron beam spectra  E 4.1.3b - The function and operation of the beam monitoring and feedback system  E 4.1.3c - How the linac controls output and dose rate for static and dynamic treatments | Oral Assessment |
|  | | LO 4.1.4 - Perform and evaluate measurements used for linac acceptance, commissioning, and routine QA, including: | | |
|  | | | E 4.1.4a - Procedures that are used for acceptance, commissioning, and ongoing QA for a linear accelerator  E 4.1.4b - Understanding linac parameters that influence tests used for acceptance, commissioning, ongoing QA and/or patient specific QA  E 4.1.4c - The dosimetric features of photon and electron beams and the physical principles and metrics for how they are assessed  E 4.1.4d - Measurement equipment requirements, uncertainties and confounding variables for tests used for acceptance, commissioning, ongoing QA and/or patient specific QA  E 4.1.4e - Connecting the observed measurement deviations for tests used with how they impact the choice of tolerances  E 4.1.4f - Comparing the differing roles of acceptance testing, commissioning and ongoing routine QA and their interrelationships | Entrustment Activity |
|  | | LO 4.1.5 - Manage a linear accelerator for clinical use, including: | | |
|  | | | E 4.1.5a – Recommending requirements for commissioning, ongoing QA programs and testing after fault repair  E 4.1.5b - Evaluating the role and function of quality systems in the linac context including periodic review, incident reporting and feedback | Entrustment Activity |
|  | *Topic 4.2 - Patient setup and immobilization, image-guided radiation therapy (IGRT) and motion management* | | | |
|  | | LO 4.2.1 - Explain the principles and aims of patient positioning, including: | | |
|  | | | E 4.2.1a - The requirements for setup reproducibility between simulation and treatment  E 4.2.1b - The clinical positioning, IGRT and motion management requirements for different anatomical sites and treatment techniques  E 4.2.1c - Considerations related to inter- and intra-fraction motion for various anatomical sites | Oral Assessment |
|  | | LO 4.2.2 - Describe the mechanisms used to ensure accurate and reproducible patient positioning, including: | | |
|  | | | E 4.2.2a - The role of patient setup lasers, the principles of their alignment and the clinical significance of misalignment  E 4.2.2b - The purpose, function, and requirements of different immobilization devices  E 4.2.2c - The physical principles and operation of imaging systems used to ensure accurate patient positioning  E 4.2.2d – The role of internal and external localization aids and motion surrogates  E 4.2.2e - The concepts, use and requirements of different motion management techniques  E 4.2.2f - The benefits and weaknesses of different patient positioning and monitoring systems | Oral Assessment |
|  | | LO 4.2.3 - Perform quality assurance procedures for patient positioning, IGRT and motion management techniques and technologies, including: | | |
|  | | | E 4.2.3a – Acceptance, commissioning, and clinical implementation of patient positioning, IGRT and motion management devices  E 4.2.3b –QA tests for patient positioning, IGRT and monitoring systems and recognizing the testing required after fault repair | Entrustment Activity |
|  | | LO 4.2.4 - Clinically apply patient positioning, IGRT and motion management strategies, including: | | |
|  | | | E 4.2.4a - Evaluating differences between systematic vs random errors for patient positioning and their relative effect on treatment delivery accuracy  E 4.2.4b - Connecting measurement deviations for QA tests with the tolerances used for patient position and monitoring systems  E 4.2.4c - Connecting IGRT and motion management strategies to the determination of clinical margins | Written Task or Report |
|  | | LO 4.2.5 - Manage patient positioning, IGRT and motion management systems, including: | | |
|  | | | E 4.2.5a - Recommending requirements for commissioning and ongoing QA programs  E 4.2.5b - Evaluating the role and function of quality systems in the patient positioning and motion management context including periodic review, incident reporting and feedback  E 4.2.5c - Understanding the parameters that influence acceptance, commissioning and ongoing QA tests of patient positioning and monitoring systems | Written Task or Report |

| **Key Area 5 – MV External Beam Treatment Planning** | | | | |
| --- | --- | --- | --- | --- |
|  | *Topic 5.1 - Basics of Treatment Planning* | | | |
|  | | LO 5.1.1 - Describe radiobiological principles for patient treatment planning, including: | | |
|  | | | E 5.1.1a - The fundamental principles of clinical radiobiology  E 5.1.1b - The use of modelling in clinical radiobiology  E 5.1.1c - The tools used to obtain radiobiological information  E 5.1.1d - The need to apply radiobiology principles to patient care | Oral Assessment |
|  | | LO 5.1.2 - Describe external beam radiation therapy treatment planning systems, including: | | |
|  | | | E 5.1.2a - The key features of an external beam radiation therapy treatment planning system  E 5.1.2b - The data requirements for treatment planning systems and how to collect, collate and curate the data for clinical use  E 5.1.2c - The requirements for planning data in the department and the management thereof  E 5.1.2d - The principles of external beam photon and electron treatment planning algorithms, including algorithms for dose and monitor unit calculation and inverse planning optimization  E 5.1.2e - The limitations and clinical relevance of dose calculation algorithms used in treatment planning  E 5.1.2f - The clinical risks and uncertainties associated with the use of the treatment planning system | Written Task or Report |
|  | | LO 5.1.3 - Practice acceptance, commissioning, and QA for an external beam radiation therapy treatment planning system, including: | | |
|  | |  | E 5.1.3a - Commissioning measurements for planning reference data  E 5.1.3b - Acceptance, commissioning, clinical implementation, and QA on an external beam radiation therapy treatment planning system | Entrustment Activity |
|  | | LO 5.1.4 - Evaluate aspects of radiation therapy treatment planning systems, including: | | |
|  | | | E 5.1.4a - Recommending requirements for commissioning and ongoing QA programs  E 5.1.4b - Evaluating the role and function of quality systems in the treatment planning context including periodic review, incident reporting and feedback  E 5.1.4c - Understanding parameters that influence acceptance, commissioning and ongoing QA tests of treatment planning systems | Oral Assessment |
|  | *Topic 5.2 - Safe and Optimal Use of Imaging* | | | |
|  | | LO 5.2.1 – Understand imaging for external beam radiation therapy treatment planning, including: | | |
|  | | | E 5.2.1a - The uncertainty associated with the use of medical images for external beam treatment planning  E 5.2.1b - Performing common operations on images used in external beam treatment planning  E 5.2.1c - Performing a series of clinical case studies to demonstrate the application of multi-modality imaging for treatment planning | Entrustment Activity |
|  | *Topic 5.3 - Clinical Application* | | | |
|  | | LO 5.3.1 - Describe the requirements of a patient treatment plan, including: | | |
|  | | | E 5.3.1a - The principles of dose prescribing and reporting in external beam treatment planning  E 5.3.1b - The principles of manual and 3D conformal MV photon external beam radiation therapy treatment planning  E 5.3.1c - The principles of IMRT/VMAT MV photon external beam radiation therapy treatment planning  E 5.3.1d - The principles of MeV electron external beam radiation therapy treatment planning | MCQ Activity |
|  | | LO 5.3.2 - Practice safe and optimal external beam radiation therapy treatment planning, including: | | |
|  | | | E 5.3.2a - Manual and 3D conformal photon external beam radiation therapy treatment planning according to established protocols  E 5.3.2b – IMRT/VMAT MV photon external beam radiation therapy treatment planning according to established protocols  E 5.3.2c - MeV electron external beam radiation therapy treatment planning according to established protocols  5.3.2d - Understand common problems that arise in the development of a treatment plan  E 5.3.2e - Evaluating clinical applications of external beam radiation therapy treatment planning systems for safe patient treatment for a variety of anatomical sites | Entrustment Activity |
|  | | LO 5.3.3 - Practice treatment planning checks, including: | | |
|  | | | E 5.3.3a - Quality control checks of individual treatment plans  E 5.3.3b - Dose/MU/time accuracy with an independent dosimetry calculation system  E 5.3.3c - Dosimetric measurements to verify the accuracy of treatment plans for individual patients - patient specific QA | Entrustment Activity |
|  | | LO 5.3.4 - Explain new, specialist, or novel treatment techniques in the department, including: | | |
|  | | | E 5.3.4a - The principles of specialist EBRT treatment techniques  E 5.3.4b - The process of implementing a new treatment technique  E 5.3.4c - The support needed for development of specialist treatment techniques | Oral Assessment |
|  | | LO 5.3.5 - Manage the quality of treatment plans, including: | | |
|  | | | E 5.3.5a - Determining recommendations for clinical application of external beam radiation therapy treatment planning systems for safe patient treatment  E 5.3.5b - Evaluating parameters that influence common problems that arise in development of a treatment plan and providing solutions for these | Entrustment Activity |

| **Key Area 6 – Superficial and Orthovoltage Therapy** | | | | |
| --- | --- | --- | --- | --- |
|  | *Topic 6.1 - Basics of Treatment Planning* | | | |
|  | | LO 6.1.1 - Describe radiation protection measures for kV treatment units, including: | | |
|  | | | E 6.1.1a - The conformance of safety systems with national or state regulations and/ or manufacturer specifications | Oral Assessment |
|  | | LO 6.1.2 - Understand and practice shielding techniques for kV treatment units, including: | | |
|  | | | E 6.1.2a - The requirements and principles of shielding construction and protection for kV treatment units  E 6.1.2b - The key concepts for shielding construction calculations for kV treatment units  E 6.1.2c - Determining barrier thicknesses and expected exposure levels for kV treatment units | Written Task or Report |
|  | *Topic 6.2 - Superficial and orthovoltage therapy equipment* | | | |
|  | | LO 6.2.1 - Describe the design of kilovoltage therapy units and the physical principles of clinical beam production, including: | | |
|  | | | E 6.2.1a - The physics of kV x-ray production including electron beam acceleration in x-ray tubes, characteristic x-rays, and bremsstrahlung radiation  E 6.2.1b - The typical angular and energy distribution of an emitted x-ray beam, including the heel effect  E 6.2.1c - How target composition, spot size and target angle, tube kV and mA, filament current and additional filtration, influence characteristics of emitted x-ray beams  E 6.2.1d - The key components of a kilovoltage therapy unit including interlocks and safety systems  E 6.2.1e - A typical superficial/orthovoltage therapy unit configuration | MCQ Activity |
|  | | LO 6.2.2 - Describe the commissioning and QA tests of a kilovoltage therapy unit, including: | | |
|  | | | E 6.2.2a - The steps in commissioning a kilovoltage therapy unit and protocols used for reference  E 6.2.2b - The relationship between commissioning tests and ongoing quality assurance practices  E 6.2.2c - How to measure relevant data, or justify the use of non-measured data, including applicator/cone/cut-out factors, shielding transmission factors, dose/ distance relationships, percentage depth dose, and back scatter factors | Written Task or Report |
|  | *Topic 6.3 - Dosimetry for superficial and orthovoltage therapy equipment* | | | |
|  | | LO 6.3.1 - Describe key kV treatment unit dosimetry protocols, including: | | |
|  | | | E 6.3.1a - The fundamental physics of dosimetry protocols, including beam quality, cross calibration, chamber choice, absorbed dose calculation, air kerma, and correction factors  E 6.3.1b - Key dosimetry protocols including selection of appropriate equipment and procedure  E 6.3.1c – Limitations, tolerances and sources of uncertainty throughout the dosimetry chain and their magnitude | Written Task or Report |
|  | *Topic 6.4 - Superficial and orthovoltage therapy planning* | | | |
|  | | LO 6.4.1 - Describe the principles of kV external beam radiation therapy treatment planning, including: | | |
|  | | | E 6.4.1a - The effects of energy, field size, field shape, beam modifiers, source to surface distance, penumbra, and normalization on kV dose distributions, including their impact on beam profile, depth dose and skin dose  E 6.4.1b - The effects of patient related factors on kV dose distributions  E 6.4.1c - Suitable materials and their thicknesses for patient shielding  E 6.4.1d – Performing treatment time or monitor-units (MUs) calculations and quality assurance for planning calculations  E 6.4.1e - The decision making influencing the choice of kV photon treatment techniques over other modalities for achieving desired dose distributions | MCQ Activity |
|  | | LO 6.4.2 - Describe kV external beam treatment planning according to established protocols, including: | | |
|  | | | E 6.4.2a - kV treatment plans that meet protocol and prescription requirements  E 6.4.2b - Strategies used to achieve acceptable plans, such as beam energy, orientation, field size, bolus, packing and shielding | Oral Assessment |

| **Key Area 7 – Imaging for Radiation Oncology** | | | | |
| --- | --- | --- | --- | --- |
|  | *Topic 7.1 - CT Imaging for Radiation Oncology* | | | |
|  | | LO 7.1.1 - Describe the physical principles and operation of CT scanners used for radiation therapy imaging, including: | | |
|  | | | E 7.1.1a - The fundamental physics of CT x-ray production  E 7.1.1b - Typical beam energies used for radiation therapy imaging  E 7.1.1c - How CT images are formed and typical CT configuration  E 7.1.1d - The principles of CT image acquisition and factors impacting on image formation and quality  E 7.1.1e - CT safety systems  E 7.1.1f – How the CT imaging plane relates to the couch movement axes and treatment isocenter | Oral Assessment |
|  | | LO 7.1.2 – Describe and practice acceptance, commissioning or QA for a CT scanner, including: | | |
|  | | | E 7.1.2a - The goals of acceptance and commissioning, general order of tests and the relevance of each major step in the procedure  E 7.1.2b - Performing tests and measurements listed in a best practice protocol and identifying limitations and tolerances  E 7.1.2c - The relationship between acceptance, commissioning, and ongoing QA tests  E 7.1.2d - The implications of differences between CT parameters and clinical use of the equipment  E 7.1.2e - Evaluating faults in major components of a CT and recognizing the tests required to return the unit to service | Entrustment Activity |
|  | | LO 7.1.3 – Understand and practice shielding techniques used for CT scanners, including: | | |
|  | | | E 7.1.3a - The requirements and principles of shielding construction and protection for CT scanners  E 7.1.3b - The key concepts for shielding construction calculations for CT scanners  E 7.1.3c - Determining barrier thicknesses and expected exposure levels for CT scanners | Written Task or Activity |
|  | *Topic 7.2 - MRI for Radiation Oncology* | | | |
|  | | LO 7.2.1 - Describe the physical principles, operation and safety of MRI systems, including: | | |
|  | | | E 7.2.1a -The fundamental physics of MRI including nuclear magnetic resonance, image formation  E 7.2.1b - The typical configuration of MRI scanners  E 7.2.1c - The key parameters and terms used in MRI  E 7.2.1d - How MRI scan settings affect image quality parameters  E 7.2.1e - Typical system-related and patient-related artifacts observed in MR images and their causes  E 7.2.1f - Comparing and contrasting the strengths and weaknesses of MR imaging  E 7.2.1g - The safety requirements for MRI systems | Oral Assessment |
|  | | LO 7.2.2 - Describe how MRI images are used in the management of cancer, including: | | |
|  | | | E 7.2.2a – How MRI is used in the management of cancer, including for diagnosis and treatment purposes  E 7.2.2b - The pros and cons of MRI in the management of cancer and at least two clinical scenarios where MRI is used  E 7.2.2c - Examples of function/spectroscopic applications to oncology  E 7.2.2d - Gated MRI techniques | Oral Assessment |
|  | *Topic 7.3 - Nuclear Medicine for Radiation Oncology* | | | |
|  | | LO 7.3.1 - Describe the basics of PET, SPECT and gamma camera systems, including: | | |
|  | | | E 7.3.1a - The fundamental physics of PET and SPECT  E 7.3.1b - Typical artifacts observed in PET/SPECT/gamma camera images and their causes  E 7.3.1c - The strengths and weakness of PET, SPECT and gamma camera imaging  E 7.3.1d - The characteristics and function of radiopharmaceuticals for diagnostic and therapeutic purposes  E 7.3.1e - Typical administered activities and patient doses | Oral Assessment |
|  | | LO 7.3.2 - Describe how PET/SPECT/gamma camera images are used in the management of cancer, including: | | |
|  | | | E 7.3.2a - How PET, SPECT and gamma camera images are used in the management of cancer  E 7.3.2b - The pros and cons of PET, SPECT and gamma camera images in the management of cancer | Oral Assessment |

| **Key Area 8 – Information and Communication Technology** | | | | |
| --- | --- | --- | --- | --- |
|  | *Topic 8.1 - Oncology Information Systems (OIS)* | | | |
|  |  | LO 8.1.1 - Describe the key design principles and operation of Oncology Information Systems, including: | | |
|  | | | E 8.1.1a - The key purpose, design principles and operation of an OIS  E 8.1.1b - The implementation planning phase for an OIS  E 8.1.1c - Electronic communication standards as used in OIS - DICOM & HL-7 E  E 8.1.1d - The impact and importance of OIS in minimizing risk  E 8.1.1e - The purpose and administration of a Record and Verify (R&V) system and its place in the wider OIS  E 8.1.1f - How data is entered into a R&V system and how it is checked  E 8.1.1g - Key features of a R&V system (e.g. tolerance tables, interlocks, user rights and warnings) | Written Task or Report |
|  | *Topic 8.2 - Data objects and types in Radiation Oncology* | | | |
|  |  | LO 8.2.1 - Describe the patient data types related to radiation therapy treatment, including: | | |
|  |  |  | E 8.2.1a - The flow of data in relation to the patient journey  E 8.2.1b - DICOM and its use in radiation therapy  E 8.2.1c – Data security and confidentiality | Written Task or Report |
|  |  | LO 8.2.2 - Explain the principle of relational database implementations within the radiation therapy process, including: | | |
|  |  |  | E 8.2.2a - Relational database implementation  E 8.2.2b - The risks in the flow of data objects between systems | Written Task or Report |
|  | *Topic 8.3 - Medical image analysis methods* | | | |
|  |  | LO 8.3.1 - Explain the theory and purpose of medical image analysis, including: | | |
|  |  |  | E 8.3.1a - Medical image analysis applications  E 8.3.1b - Common medical image analysis techniques | Written Task or Report |
|  | *Topic 8.4 - Software automation and artificial intelligence (AI) basics* | | | |
|  |  | LO 8.4.1 - Explain software automation and AI applications in clinical practice, including: | | |
|  |  |  | E 8.4.1a - The clinical application of software automation and AI  E 8.4.1b - Common software automation techniques  E 8.4.1c - The pros and cons of in-house and multi-center validation of radiomics features with standard approaches | Written Task or Report |
|  |  | LO 8.4.2 - Describe big data and enterprise imaging, including: | | |
|  |  |  | E 8.4.2a - The clinical application of big data and enterprise imaging (EI)  E 8.4.2b - The pros and cons of in-house and multi-center validation of big data and EI with standard approaches | Written Task or Report |
|  |  | LO 8.4.3 - Compare and contrast the quality, regulatory, and ethical issues of data utilization, with the advantages of automation, software development and AI processes, including: | | |
|  |  |  | E 8.4.3a - AI device management  E 8.4.3b - Post-market surveillance of a CE marked device  E 8.4.3c - Health technology assessment (HTA) of an AI device | Written Task or Report |

| **Key Area 9 – Brachytherapy** | | | | |
| --- | --- | --- | --- | --- |
|  | *Topic 9.1 - High dose rate (HDR) brachytherapy* | | | |
|  | | LO 9.1.1 - Explain radiation safety and protection as it relates to radioactive sources, including: | | |
|  | | | E 9.1.1a - Legislative requirements for management of radioactive sources  E 9.1.1b - Emergency procedures  E 9.1.1c - Regulatory and safety requirements for clinical treatment  E 9.1.1d - The requirements and principles of shielding construction and protection for HDR brachytherapy treatment rooms  E 9.1.1e - The key concepts for shielding construction calculations for HDR brachytherapy treatment rooms  E 9.1.1f - Determining barrier thicknesses and expected exposure levels for HDR brachytherapy treatment rooms | Oral Assessment |
|  | | LO 9.1.2 - Explain HDR brachytherapy as a treatment modality, including: | | |
|  | | | E 9.1.2a - Patient selection for HDR treatments, instead of, or in combination with external beam radiation therapy (EBRT)  E 9.1.2b - HDR treatment regimes  E 9.1.2c - HDR treatment sites  E 9.1.2d - Radiobiological equivalence of treatment schemes, including combined EBRT - HDR brachytherapy treatment and correctly use radiobiological calculation methods. Describe the limitations. | Written Task or Report |
|  | | LO 9.1.3 - Describe clinical HDR delivery systems, including: | | |
|  | | | E 9.1.3a - The physics principles of HDR sources  E 9.1.3b - The design principles and operation of HDR systems  E 9.1.3c - Acceptance, commissioning, and QA tests on an HDR system | Written Task or Report |
|  | | LO 9.1.4 - Describe source strength determination methods, including: | | |
|  | | | E 9.1.4a - The traceability of calibration factors for chambers used in brachytherapy  E 9.1.4b - The principles of the protocol used for source strength determination in a department | Written Task or Report |
|  | | LO 9.1.5 - Describe the clinical use of HDR treatment planning systems, including: | | |
|  | | | E 9.1.5a - The physics principles of brachytherapy treatment planning systems  E 9.1.5b - The commissioning and QA requirements for an HDR brachytherapy treatment planning system  E 9.1.3c - Principles of HDR brachytherapy treatment planning  E 9.1.3d - The operational process in developing a treatment plan for brachytherapy  E 9.1.3e - Plan checks on HDR brachytherapy treatment plans  E 9.1.3f - The uncertainties involved in HDR brachytherapy planning and delivery | Written Task or Report |
|  | | LO 9.1.6 - Explain the use of imaging systems for applicator insertion and treatment planning, including: | | |
|  | | | E 9.1.6a - The principles of imaging modalities used for HDR brachytherapy  E 9.1.6b - The selection of imaging modalities used for HDR brachytherapy | Oral Assessment |
|  | *Topic 9.2 - Low dose rate (LDR) brachytherapy* | | | |
|  | | LO 9.2.1 - Explain the fundamental principles of LDR brachytherapy, including: | | |
|  | | | E 9.2.1a - The physics principles of LDR brachytherapy sources  E 9.2.1b - LDR treatment regimes  E 9.2.1c - Principles of LDR brachytherapy treatment planning  E 9.2.1d - Principles of ultrasound imaging and its use in LDR brachytherapy  E 9.2.1e - The principles and practices of LDR brachytherapy source handling  E 9.2.1f - The principles and practices of LDR source calibration and quality management | Written Task or Report |

| **Key Area 10 – Advanced Technologies** | | | | |
| --- | --- | --- | --- | --- |
|  | *Topic 10.1 - Proton Therapy* | | | |
|  | | LO 10.1.1 - Describe the principles of proton therapy, including: | | |
|  | | | E 10.1.1a - The clinical rationale for the use of proton compared to photon radiation therapy  E 10.1.1b - Key clinical evidence for the use of proton therapy  E 10.1.1c - The role of clinical trials in providing clinical evidence for proton therapy  E 10.1.1d - Different methods for selecting patients likely to benefit from proton therapy  E 10.1.1e - The cost vs clinical benefit for proton therapy | Online Assessment |
|  | | LO 10.1.2 - Explain proton/heavy ion physics and proton/heavy ion dosimetry, including: | | |
|  | | | E 10.1.2a - The physics of proton and heavy ion interactions in biological tissues and other relevant materials  E 10.1.2b - How Linear Energy Transfer (LET) and Radiobiological Effectiveness (RBE) influence proton and heavy ion radiation therapy dose distributions  E 10.1.2c - Secondary nuclear fragments in heavy ion therapy and how they affect the physical and biological dose distributions  E 10.1.2d - The RBE and LET distribution of a proton and heavy ion depth dose curve and how they are accounted for in treatment planning systems  E 10.1.2e - The dosimetry protocols in use for proton and heavy ion therapy | Online Assessment |
|  | | LO 10.1.3 - Describe proton beam delivery systems, including: | | |
|  | | | E 10.1.3a - The fundamental components of a clinical proton therapy facility  E 10.1.3b - The principle of operation of cyclotrons and synchrotrons and the benefits and limitations of each  E 10.1.3c - The main components of clinical proton therapy gantries and their role in generating a clinical proton beam  E 10.1.3d - Passive scattering and pencil beam scanning beam generation and the pros and cons of each | Online Assessment |
|  | | LO 10.1.4 - Describe basic proton beam treatment planning, including: | | |
|  | | | E 10.1.4a - Particle therapy dose calculation methods  E 10.1.4b - The causes and clinical implications of range uncertainties and strategies to mitigate them  E 10.1.4c - Methods of optimization and evaluation of dose distributions  E 10.1.4d - The challenges of equitable comparison of photon and proton plans | Online Assessment |
|  | *Topic 10.2 - MRI Linacs* | | | |
|  | | LO 10.2.1 – Explain fundamental physical principles as it relates to the operation of MRI linacs, including: | | |
|  | | | E 10.2.1a - The key design principles and operation of an MRI linac  E 10.2.1b - Basic MRI linac safety principles  E 10.2.1c - Conventional imaging system for a linac vs an MRI linac | Online Assessment |
|  | | LO 10.2.2 - Explain MRI linac physics as it relates to the fundamentals of dosimetry, including: | |  |
|  | | | E 10.2.2a - Changes in fundamental linac dosimetry within an MRI environment  E 10.2.2b - MRI linac quality assurance principles | Online Assessment |
|  | | LO 10.2.3 - Explain MRI linac treatment planning, including: | |  |
|  | | | E 10.2.3a - Methods of online and offline treatment plan adaptation  E 10.2.3b - Patient and treatment aid modelling for MRI-guided RT compared to conventional IGRT  E 10.2.3c - Key considerations of planning QA for MRI-guided RT and adaptive RT  E 10.2.3d - Methods used to reduce intra-fraction motion on MRI linacs | Online Assessment |
